# Magnesium may be a key nutrient mechanism related to Fusarium wilt resistance: a new banana cultivar (Zhongjiao No. 9)

**DOI:** 10.7717/peerj.11141

**Published:** 2021-04-05

**Authors:** Weifang Hu, Baomei Yang, Zhaohuan He, Guoliang Li

**Affiliations:** Institute of Agricultural Resources and Environment, Guangdong Academy of Agricultural Sciences, Guangzhou, China

**Keywords:** Banana, Nutrient budgets, Nutrient mechanism, Fusarium wilt-resistant, Zhongjiao no.9

## Abstract

Zhongjiao No. 9 (*Musa* spp.), a new Fusarium wilt-resistant banana cultivar, has shown considerable promise in the field. However, the growth, nutrient budgets, and key nutrient mechanisms related to Fusarium wilt resistance have not been explicitly examined. Here, the plant growth, yield, fruit quality, and nutrient budgets of Zhongjiao No. 9 were investigated. The results showed that Zhongjiao No. 9 has a large biomass with a high yield (54.65 t ha^−1^). The concentrations of N, P, K, Ca, Mg, Mn, B, and Mo were mainly high in the leaves and bunches of mother plants as well as in the leaves and pseudostems of daughter plants, while Cu and Fe were enriched in the roots of both mother plants and daughter plants. Linear discriminant analysis revealed that K, Ca, and Fe were important for plant growth in both the mother plants and daughter plants; S, Zn, and Mn were important for the mother plants, and N, P, and B for were important for the daughter plants. The nutrient uptake ratio of N:P:K:Ca:Mg:S was 1:0.13:3.86:0.68:0.40:0.07. Compared with local cultivars, there was a higher Mg concentration in pseudostems and a higher Mg uptake ratio were observed in Zhongjiao No. 9. Together, our results provide insight into the importance of Mg accumulation in relation to Fusarium wilt resistance, and we provide information on nutrient demands and fertilization application.

## Introduction

Bananas (genus Musa, family Musaceae) are among the most widely consumed foods in the world (Rossmann et al., 2012). Globally, over 100 Mt of bananas are grown annually on an estimated area of approximately 5 million ha, with production concentrated in the tropics and subtropics, and the extent of the area suitable for banana cultivation is predicted to nearly double by the 2060s due primarily to climatic drying (Machovina & Feeley, 2013). However, there are many diseases that severely limit banana yields, and *Fusarium* wilt (Panama disease) is one of the most destructive soil-borne diseases in banana orchards worldwide and is caused by *Fusarium oxysporum* f. sp. *cubense* (Foc) (Jain, 2005; Ploetz, 2015; Liu et al., 2019). To date, several control key strategies have been proposed in the disease suppression of banana *Fusarium* wilt, including physical and chemical controls (Domınguez, Negrın & Rodrıguez, 2001; Domınguez, Negrın & Rodrıguez, 2003; Fortunato et al., 2012; Li et al., 2018), and biological controls (Nel et al., 2006; Zhang et al., 2011; Ho et al., 2015; Shen et al., 2015a; Shen et al., 2015b; Wang et al., 2016;  Gómez Expósito et al., 2017). Despite the substantial, positive literature on these topics, the complexity of Fusarium wilt remains elusive (Ploetz, 2015). Thus, the development of disease-resistant banana varieties is necessary for replacing susceptible cultivars in infested soils (Xu et al., 2011; Ploetz, 2015; Zuo et al., 2018). However, a lack of knowledge about plant nutrient budgets and disease-resistant varieties is a large hindrance to managing banana plantations.

A new Fusarium wilt-resistant banana cultivar, Zhongjiao No. 9 (*Musa* spp.), was introduced by the Institute of Fruit Tree Research, Guangdong Academy of Agricultural Sciences, China in 2016. It has shown considerable promise in the field, and the cultivar not only has high resistance to Fusarium wilt but also has a high yield performance. For healthy growth, soil nutrients and plant budgets must be surveyed; and the efficient use of nutrients is an effective way to ensure high and stable crop productivity (Bom, Magid & Jensen, 2019). Understanding the partial dry matter and nutrient balances in banana plants associated with soil nutrients can provide options for optimum productivity and nutrient management as well as minimum resource wastes (Ndabamenye et al., 2013b; Zhang et al., 2019b). To our knowledge, the growth and nutrient budgets of Zhongjiao No. 9 have not been explicitly examined and thus management strategies remains lacking. Nonetheless, uninformed fertilization may not meet the nutrient demands of banana plants that are necessary for growth and can easily cause pollution. Additionally, previous studies have demonstrated that some nutrient elements in banana plants and their associated soils can reduce the severity of plant diseases by increasing disease tolerance and resistance against plant pathogens (Dordas, 2008; Siddiqui et al., 2015; Wu et al., 2020). Other studies have demonstrated that the manipulation of soil nitrogen (N), phosphorus (P), potassium (K), magnesium (Mg), manganese (Mn), and zinc (Zn) levels may reduce the incidence of Fusarium disease by direct toxic effects or by affecting the cell wall composition, lignin biosynthesis, phenol biosynthesis, photosynthesis, and several other functions (Yergeau et al., 2006; Dordas, 2008; Hassan & Abo-Elyousr, 2013; Wu et al., 2020). However, few studies have focused on the nutrient budgets of the *Fusarium* wilt-resistant cultivar described above, and particularly, the key nutrient mechanism related to Fusarium wilt-resistant remains uncertain. We hypothesized that the concentrations and budgets of N, P, K, Mg, Mn, and Zn may be higher Zhongjiao No. 9 plants than in other local banana varieties. Therefore, we compared the nutrient budgets of Zhongjiao No. 9 with those of previously reported varieties, including plantain (*Musa ABB* Pisang Awak), Giant Cavendish cv. Baxi (*Musa AAA,* hereinafter called Baxi), and Williams B6 (*Musa* AAA Cavendish) (Yang et al., 2013; Yao et al., 2005; Zhang et al., 2019a). In this study, we investigated the plant growth, yield, fruit quality, and nutrient budgets of Zhongjiao No. 9. We aimed to (i) elucidate the physiological characteristics of the plants, (ii) identify the nutrients that are key for plant growth and the key nutrient mechanisms related to Fusarium wilt resistance, and (iii) provide a theoretical basis for the fertilization of Zhongjiao No. 9.

## Material and Methods

### Site description

**Figure 1:**
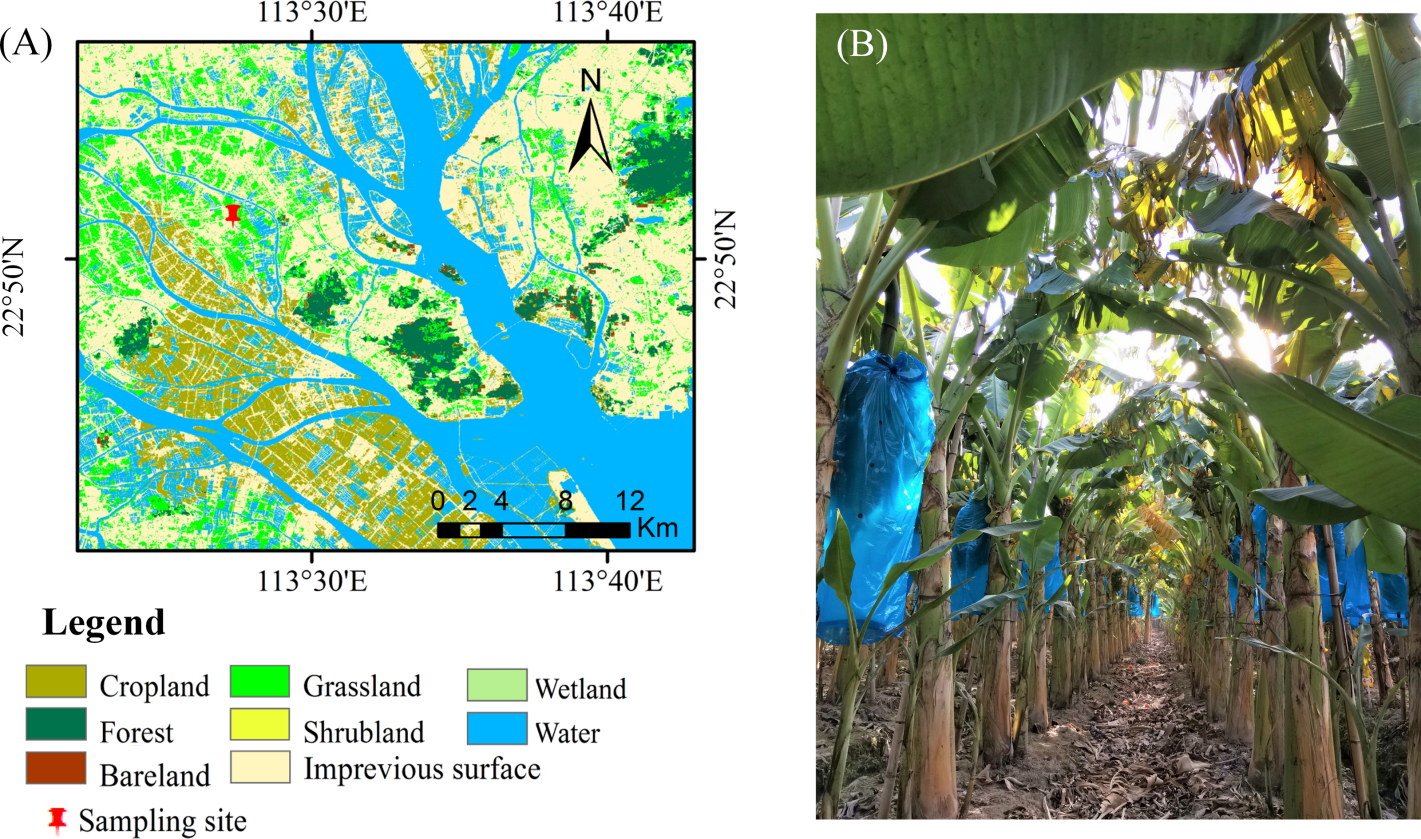
Positioning of study sites in southeastern China. (B) The plantation of the Zhongjiao NO.9.

This study was conducted in the suburbs of Guangzhou, China (22°53′30.95″N, 113°25′45.99″, Fig. 1). The region has a typical subtropical humid monsoon climate. The annual average temperature and precipitation range from 21.4–21.9 °C and 1623.6–1899.8 mm, respectively. The soil is predominantly loamy clay with acidic conditions (pH = 4.19, 1:2.5 sediment: water suspension) (Ndabamenye et al., 2013a). The properties of the soil (5 subsamples per plot, randomly selected) at the 0–30 cm around each selected plant were determined at harvest, covering differences in soil fertility levels, from areas close to the homesteads to the borders of the outer fields (Ndabamenye et al., 2012). These soil properties are shown in Table 1.

### Plant material and growth conditions

Banana seedlings of Zhongjiao No. 9 were provided by the Institute of Fruit Tree Research, Guangdong Academy of Agricultural Sciences, China. Zhongjiao No.9 were obtained by government approval (Fruit Variety Certification of the Guangdong, 20170001, http://dara.gd.gov.cn/zzglc/content/post_1567853.html). The banana seedlings were provided by the Institute of Fruit Tree Research, Guangdong Academy of Agricultural Sciences, China, and these seedlings are kept in their seed stock centers. The planting density was 2077 plants ha^−1^ with a basal dressing of 613.6 kg N ha^−1^, 613.6 kg K_2_O ha^−1^, and 763.6 kg P_2_O_5_ ha^−1^ applied to each planting plot. The applied fertilizers were compound fertilizers (Yara Mila (15-15-15, Norway), Azomureş (15-15-15, Romanian)], and Kaliumsulfat (K+S KALL GmbH, 50% K_2_O, Germany)). Thereafter, the pruned leaves were left as mulch in the plots.

**Table 1:** The soil properties in the study sites.

Parameters	Mean ± Standard Error	Min.	Max.
pH	4.19 ± 0.04	4.09	4.29
SOM (g kg^−^^1^)	16.69 ± 0.36	15.78	17.61
Hydrolysates N (mg kg^−^^1^)	92.12 ± 2.69	83.68	97.51
Available P (mg kg^−^^1^)	202.03 ± 20.30	155.34	263.77
Available K (mg kg^−^^1^)	154.72 ± 25.01	105.65	249.68
Exchangeable Ca (mg kg^−^^1^)	636.63 ± 43.30	2.65	3.88
Exchangeable Mg (mg kg^−^^1^)	167.86 ± 13.48	1.15	1.76
Available S (mg kg^−^^1^)	44.98 ± 4.32	31.38	56.70
Available Cu (mg kg^−^^1^)	6.67 ± 0.10	6.42	6.88
Available Zn (mg kg^−^^1^)	6.66 ± 0.15	6.33	7.16
Available Fe (mg kg ^−^^1^)	175.16 ± 9.74	143.01	199.33
Available Mn (mg kg^−^^1^)	65.33 ± 2.94	61.63	76.98
Available B (mg kg^−^^1^)	0.52 ± 0.05	0.41	0.63
Available Mo (mg kg^−^^1^)	0.19 ± 0.01	0.18	0.20

### Sampling and analysis

Samples were randomly taken from five different healthy plants in the maturity stage (June 19, 2018). The height, total number of leaves, and circumference of the pseudostems of each plant were determined. The banana plants, including mother plants (plant crops) and daughter plants (ratoon crops), were completely removed at harvest. The fresh weights of the mother plants (leaves, pseudostems, corms, roots, bunches, and fingers) and the daughter plants (leaves, pseudostems, corms, and roots) were recorded and then sampled using the quartering method.

The collected plant samples were carefully rinsed with deionized water and then weighed after being oven-dried at 105 °C for 48 h. Then, the dried samples were ground and passed through a 0.149-mm sieve and retained for further chemical analyses. The samples were digested with H_2_SO_4_-H_2_O_2_, and then the total N, P, and K concentrations in the digested solutions were determined using the distillation method with an automatic Kjeldahl apparatus (Kjeltec 8200, FOSS, Denmark), molybdenum antimony impedance colorimetry with a spectrophotometer (V-5100, Yuanxi, Shanghai, China), and atomic absorption spectrophotometry (ZA3300, Hitachi, Tokyo, Japan), respectively (Lu, 1999). Furthermore, the samples were digested with HNO_3_-HClO_4_, and the calcium (Ca), iron (Fe), copper (Cu), Mg, Mn, and Zn concentrations in the digest solutions were determined using atomic absorption spectrophotometry (Lu, 1999). The total sulfur (S) content was determined with nephelometry by a spectrophotometer after digestion with HNO_3_-HClO_4_ (Lu, 1999). Additionally, the boron (B) and molybdenum (Mo) concentrations were determined using curcuma colorimetry and polarographic adsorptive complex catalytic waves after dry ashing, respectively (Lu, 1999). All elements were verified with the plant standard reference material (GBW07603 and GB07408, China).

The number of hands and the total number of fingers per bunch were counted, and the lengths and weights of the fingers were measured. The proportion of edible fruits was calculated. The bottom row of the third hand of each bunch was selected with which to determine the quality characteristics when the fruits were naturally and uniformly ripened, at the time the percent total soluble solids, total sugar, and ascorbic acid were determined (Kavino et al., 2010).

### Data analysis

One-way analysis of variance (ANOVA) was used to determine the difference among different organs, two-factor ANOVA was used to determine the difference between mother plants and daughter plants, and the probability level used to determine significance was *p* < 0.05. Linear discriminant analysis (LDA) by the stepwise method was carried out to evaluate the key nutrients in the plants (Ma et al., 2016). Pearson correlation analyses were performed to assess the relationships among soil parameters, plant nutrients, and fruit yield and quality. The above statistical analysis was performed using SPSS 22.0 (SPSS. Inc., Chicago, IL, USA). Plots were performed using Origin 9.3 (OriginLab Corporation, Northampton, MA, USA).

## Results

### Plant growth, yield, and quality characteristics

When the Zhongjiao No. 9 plants were completely removed at harvest, their average height and girth were 292 cm and 74 cm, respectively, with an average yield of 54.65 ±1.39 t ha^−1^ (Table 2). The highest and lowest dry mass were found in pseudostems and roots, respectively. In addition, the average percentages of total soluble solids, total sugar, and ascorbic acid were 21.60%, 208.08 mg g^−1^, and 3.40 mg per 100 g fresh fruit, respectively (Table 2).

**Table 2:** The plant morphological, fruit, and biomass characteristics in the maturity stages.

Morphological characteristics			Fruit characteristics		Biomass characteristics				
					Organ	Fresh weight (kg plant^−^^1^)		Percentage of total dry mass (%)	
						Plant crop	Ratoon crop	Plant crop	Ratoon crop
Plants	Height (cm)	292	Proportion of edible fruits (%)	63.99	Leaves	8.78	1.68	15.24	31.63
	Girth (cm)	74			Pseudostems	38.08	7.65	20.70	33.74
	Total number of leaves	8	Total soluble solids (%)	21.60	Corms	16.86	2.88	16.69	31.44
Fingers	Hands/ bunch	16	Ascorbic acid (mg 100 g^−1^)	3.40					
	Fingers/ bunch	118	Total sugar (mg g^−1^)	208.08	Roots	2.14	0.30	1.62	3.19
	Finger length (cm)	24.97			Bunches	3.16	n.a.	1.81	n.a.
	Finger circumference (cm)	14.72			Fingers	26.31	n.a.	43.94	n.a.
	Finger weight (kg)	0.22							
	Yield (t ha^−^^1^)	54.65							

**Notes.**

Values are the means of five replicates.

n.a. data not available

### Distribution of nutrient concentrations

**Figure 2:**
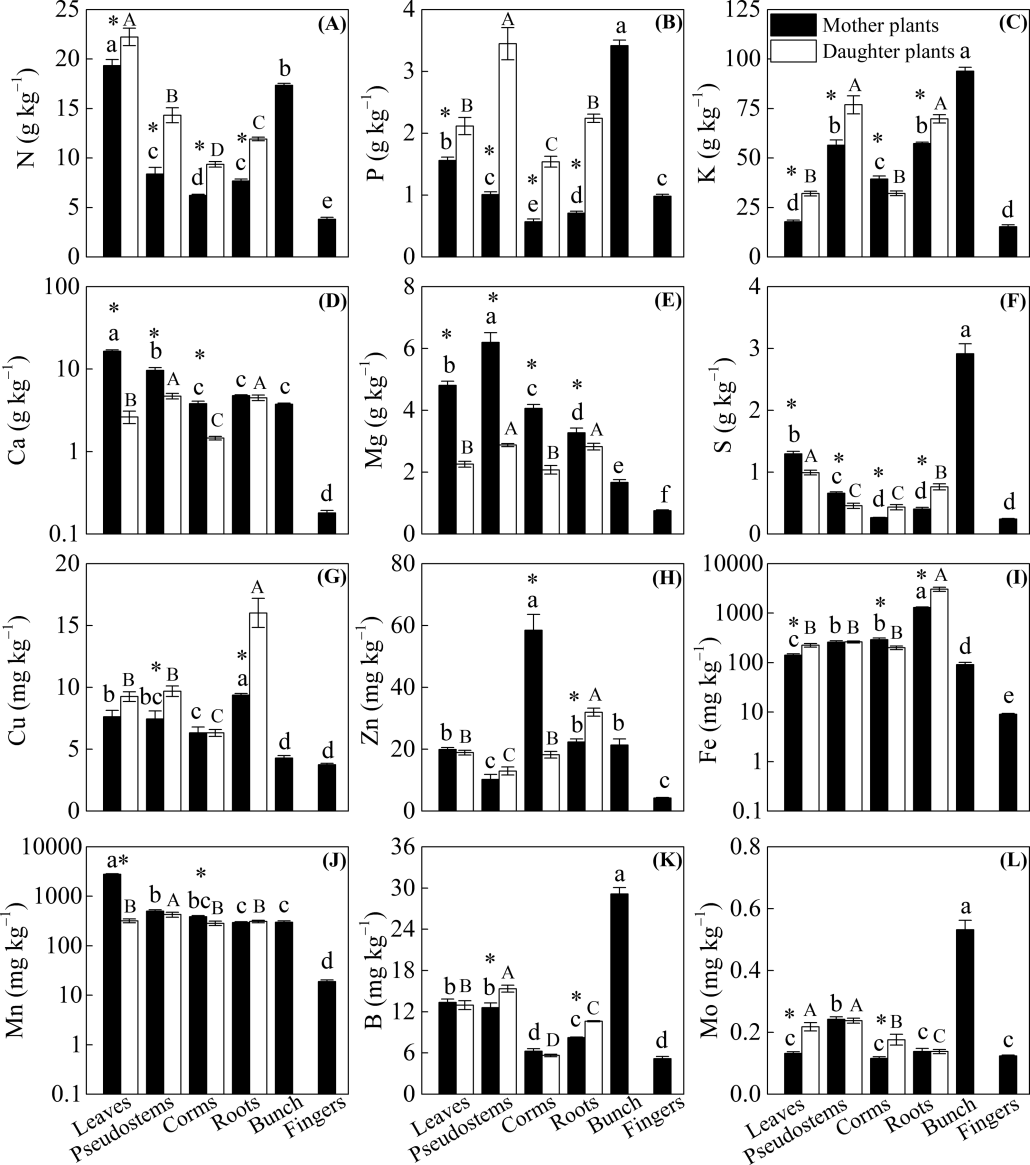
The distribution of (A) N, (B) P, (C) K, (D) Ca, (E) Mg, (F) S, (G) Cu, (H) Zn, (I) Fe, (J) Mn, (K) B, and (L) Mo in different organs of the mother plants and daughter plants (mean ± standard error). Lowercase letters above bars denote a significant difference among different organs of mother plants (*p* < 0.05), and capital letters above bars denote a significant difference among different organs of daughter plants (*p* < 0.05), while an ^*^ above bars the denotes a significant difference between mother plants and daughter plants (*p* < 0.05).

Figure 2 shows that significantly lower N, P, and K concentrations and higher Ca and Mg concentrations were observed among the leaves, pseudostems, corms, and roots of mother plants than in daughter plants (*p* < 0.05), with the exception of a higher K concentration observed in the corms of mother plants and no significant difference in Ca in the roots of mother or daughter plants. Compared with the daughter plants, we found higher S and Mn levels in leaves, higher S levels in pseudostems, and higher Fe and Mn levels in corms. However, lower Mo levels in leaves, Cu and B levels in pseudostems, S and Mo levels in corms, and S, Cu, Zn, Fe, and B levels in roots were observed in mother plants than in daughter plants (*p* < 0.05, Fig. 2).

The concentrations of N, P, K, Ca, Mg, and Mn were mainly enriched in the leaves and bunches of mother plants, as well as in the leaves and pseudostems of daughter plants, whereas Cu and Fe were enriched in the roots of both mother plants and daughter plants (*p* < 0.05, Fig. 2). In mother plants, significantly higher concentrations of S, B, and Mo were found in the bunches, while a higher Zn concentration in corms and a higher Mn concentration in leaves were observed (*p* < 0.05, Fig. 2). In addition, most of the nutrient concentrations were lowest in the fingers of mother plants, whereas the lowest nutrients in daughter plants were found in the corms (*p* < 0.05, Fig. 2).

**Figure 3:**
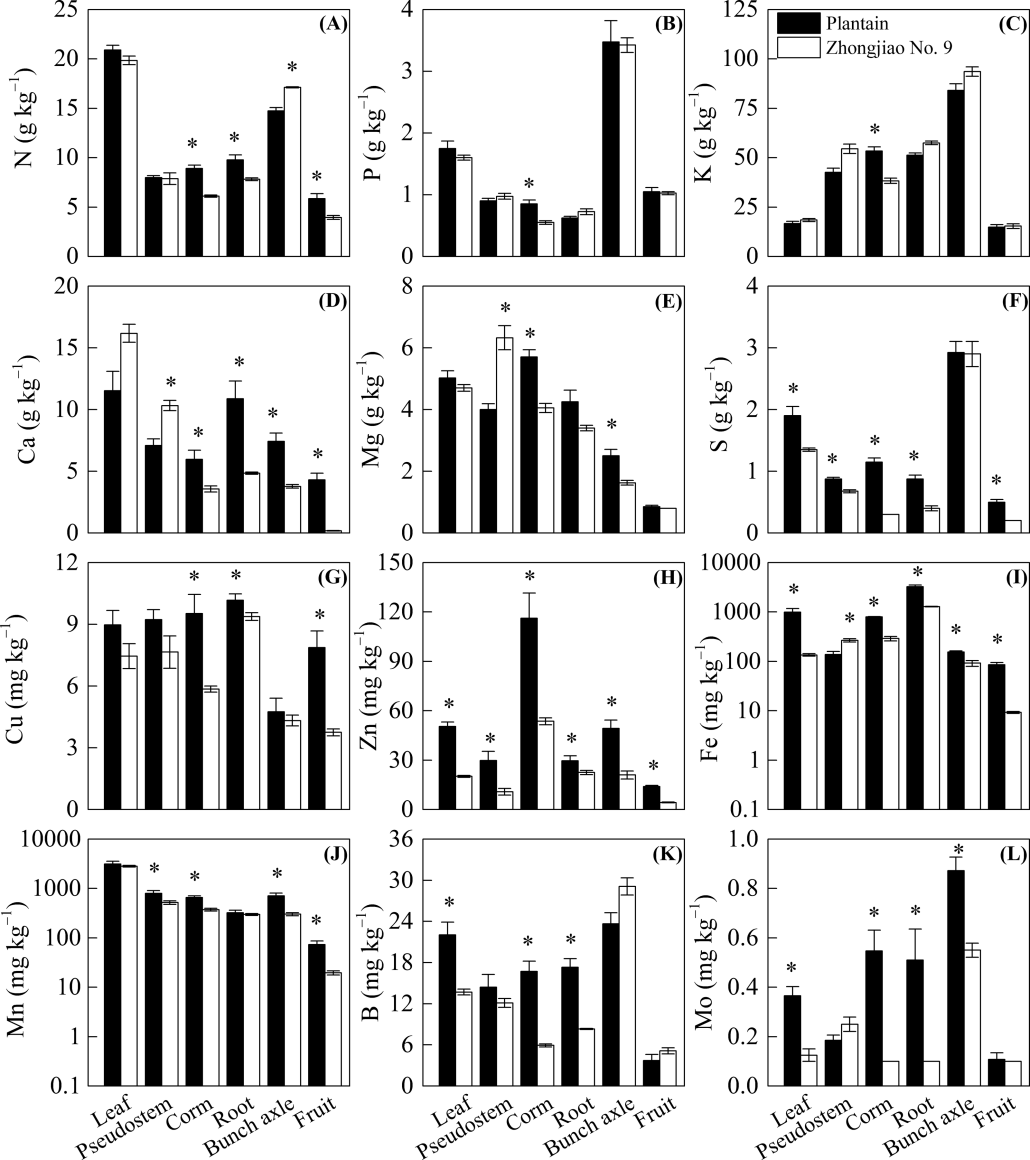
Concentration of (A) N, (B) P, (C) K, (D) Ca, (E) Mg, (F) S, (G) Cu, (H) Zn, (I) Fe, (J) Mn, (K) B, and (L) Mo in the mother plants of the Zhongjiao NO.9 and the plantain. Mean ± standard error. ^*^ above bars the denotes a significant difference between the Zhongjiao NO.9 and the plantain (*p* < 0.05).

Compared with the plantain plants, all the measured nutrients were significantly lower in Zhongjiao No. 9. The S, Zn, Fe, B, and Mo levels in leaves as well as the N, Ca, S, Cu, Zn, Fe, B, and Mo levels in roots were lower in Zhongjiao No. 9 than in plantain (Fig. 3). There were significantly higher Ca, Mg, and Fe levels as well as lower S, Zn, and Mn levels observed in the pseudostems of Zhongjiao No. 9 compared with those observed in plantain (Fig. 3). There were higher N and lower Ca, Mg, Zn, Fe, Mn, and Mo levels in bunches as well as lower N, Ca, S, Cu, Zn, Fe, and Mn levels in the fruits of Zhongjiao No. 9 than those in the fruits of plantain (Fig. 3). It is worth noting that the Ca concentration was approximately 24 times higher in the fruits of plantain than with those of Zhongjiao No. 9.

### Nutrient budgets and outputs

In the mother plants, the highest enrichment of N, Ca, S, and Mn in leaves, considerably higher K, Mg, B, and Mo stocks in pseudostems, more Zn budgets in corms, significantly higher P stocks in fingers, similar and considerably higher Fe stocks in pseudostems and corms, and significantly higher Cu and Mo budgets in pseudostems and fingers were observed (*p* < 0.05, Table 3). In contrast, most of the nutrient budgets were much lower in the roots and/or bunches of mother plants (*p* < 0.05, Table 3). In the daughter plants, most of the nutrient stocks were considerably higher in the leaves but lower in the pseudostems, with the exception of higher Fe budgets in the roots (*p* < 0.05, Fig. 3 and Table 3). Overall, the total nutrient budgets of the mother plants were calculated as follows: 85.05 g N plant^−1^, 11.38 g P plant^−1^, 328.33 g K plant^−1^, 58.02 g Ca plant^−1^, 33.72 g Mg plant^−1^, 5.97 g S plant^−1^, 60.89 mg Cu plant^−1^, 187.66 mg Zn plant^−1^, 1631.05 mg Fe plant^−1^, 6583.95 mg Mn plant^−1^, 93.54 mg B plant^−1^, and 1.67 mg Mo plant^−1^.

Compared with the plantain, Baxi, and Williams B6, most nutrient budgets were relatively low in the roots and fingers of Zhongjiao No. 9, and most nutrient budgets in the leaves and corms of Zhongjiao No. 9 were between those of plantain and Baxi (Table 4). The nutrient uptake ratios of N:P:K:Ca:Mg:S of Zhongjiao No. 9, plantain, and Baxi were 1:0.13:3.86:0.68:0.40:0.07, 1:0.12:3.12:0.71:0.33:0.10, and 1:0.90:3.72: 0.55:0.27:0.09, respectively. To compare the aboveground nutrient uptake levels at the same yield values, we calculated the nutrient uptake levels at a high yield of 60 t ha^−1^ (Table 5). Compared with Zhongjiao No. 9, all nutrients had larger stored in plantain and Baxi, with the exception of the medium levels of Fe and Mn uptake observed in Zhongjiao No. 9 (Table 5).

### Relationships among soil-plant-fruit nutrients

**Figure 4:**
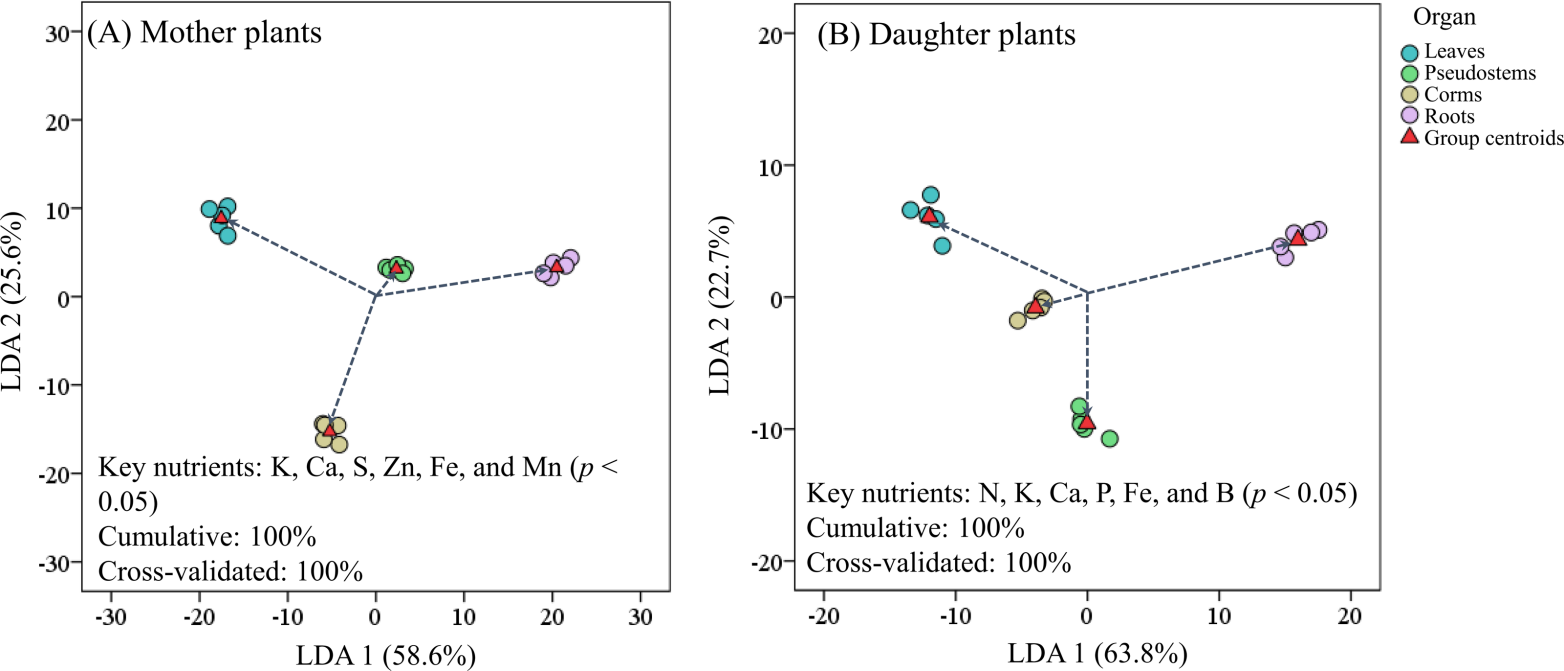
Scatter plot of (A) mother plants and (B) daughter plants nutrients in different organs based on linear discriminant analysis with a stepwise discriminant procedure.

**Figure 5:**
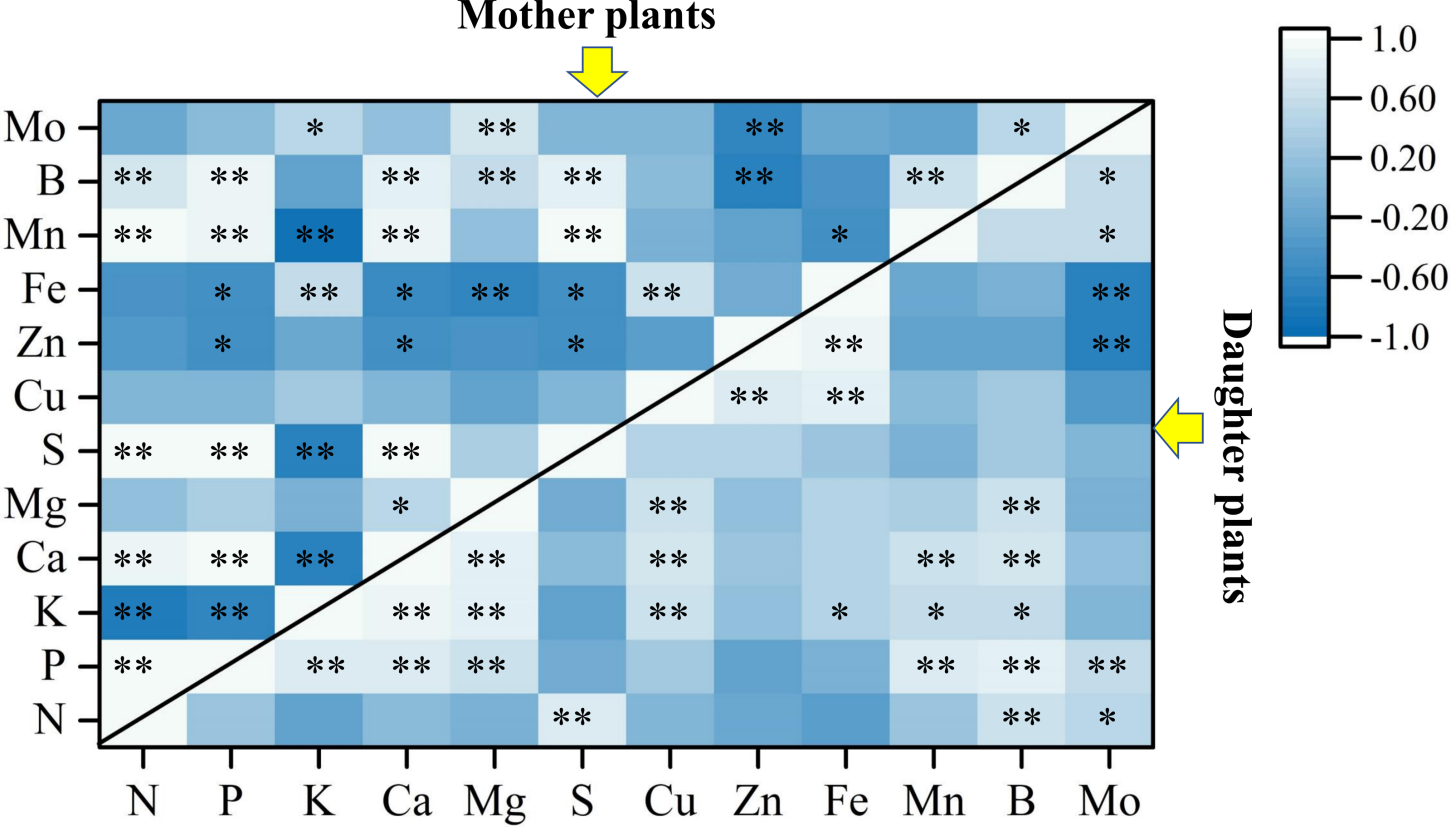
Pearson correlation analyses among plant nutrients in mother plants and daughter plants. The asterisks ^*^ and ^*^^*^ indicate significant correlations at the 0.05 and 0.01 levels, respectively.

Figure 4 revealed that the LDA models with stepwise discriminant procedures had high recognition (100% of cumulative) and satisfactory predictive ability (100% of cross-validated) for classifying plant nutrient samples on the basis of Mahalanobis distance values. The K, Ca, and Fe were important in both mother plants and daughter plants, while S, Zn, and Mn were important in mother plants, and N, P, and B were important in daughter plants (Fig. 4). Pearson correlation analyses further showed that these key nutrients were significantly correlated with other unselected nutrients (Fig. 5).

**Table 3:** Nutrient budgets in different organs of the plant crops and ratoon crops.

Nutrients	Leaves		Pseudostems		Corms		Roots		Bunches	Fingers
	Mother plants	Daughter plants	Mother plants	Daughter plants	Mother plants		Daughter plants	Mother plants		Daughter plants	Mother plants	Mother plants
N (g plant^−1^)	31.93 a^*^	3.19 A	18.61 b^*^	0.47 C	11.22 c^*^		0.76 BC	1.35 d^*^		0.88 B	3.77 d	18.18 b
P (g plant^−1^)	2.57 b^*^	0.30 A	2.25 c^*^	0.11 C	1.02 d^*^		0.13 C	0.13 e^*^		0.17 B	0.74 d	4.67 a
K (g plant^−1^)	29.17 c^*^	4.57 A	125.69 a^*^	2.54 C	70.32 b^*^		2.62 C	10.08 d^*^		5.13 B	20.34 c	72.73 b
Ca (g plant^−1^)	27.15 a^*^	0.38 A	21.65 b^*^	0.16 B	6.72 c^*^		0.12 B	0.84 d^*^		0.33 A	0.81 d	0.86 d
Mg (g plant^−1^)	7.93 b^*^	0.32 A	13.99 a^*^	0.10 D	7.27 b^*^		0.17 C	0.58 d^*^		0.21 B	0.36 d	3.59 c
S (g plant^−1^)	2.15 a^*^	0.14 A	1.49 b^*^	0.01 D	0.48 d^*^		0.03 C	0.07 e^*^		0.06 B	0.63 d	1.15 c
Cu (g plant^−1^)	12.67 b^*^	1.32 A	16.63 a^*^	0.32 C	11.24 b^*^		0.52 B	1.65 c^*^		1.17 A	0.93 c	17.78 a
Zn (g plant^−1^)	32.98 b^*^	2.70 A	22.48 c^*^	0.43 C	103.73 a^*^		1.49 B	3.92 d^*^		2.35 A	4.61 d	19.94 c
Fe (g plant^−1^)	234.36 b^*^	31.64 B	576.79 a^*^	8.66 B	528.13 a^*^		16.16 B	228.80 b		223.99 A	19.78 c	43.19 c
Mn (g plant^−1^)	4590.71 a^*^	45.64 A	1104.12 b^*^	14.05 C	682.47 c^*^		23.47 B	51.96 d^*^		22.71 B	64.69 d	90.00 d
B (g plant^−1^)	22.06 b^*^	1.85 A	27.99 a^*^	0.51 C	11.15 c^*^		0.46 C	1.46 e^*^		0.78 B	6.30 d	24.58 b
Mo (g plant^−1^)	0.22 b^*^	0.03 A	0.54 a^*^	0.01 C	0.20 b^*^		0.02 B	0.02 d^*^		0.01 C	0.11 c	0.57 a

**Notes.**

Values are the means of five replicates. Lowercase letters denote a significant difference among different organs in plant crops (*p* < 0.05), and capital letters denote a significant difference among different organs ratoon crops (*p* < 0.05).

* A significant difference between plant crops and ratoon crops (*p* < 0.05).

**Figure 6:**
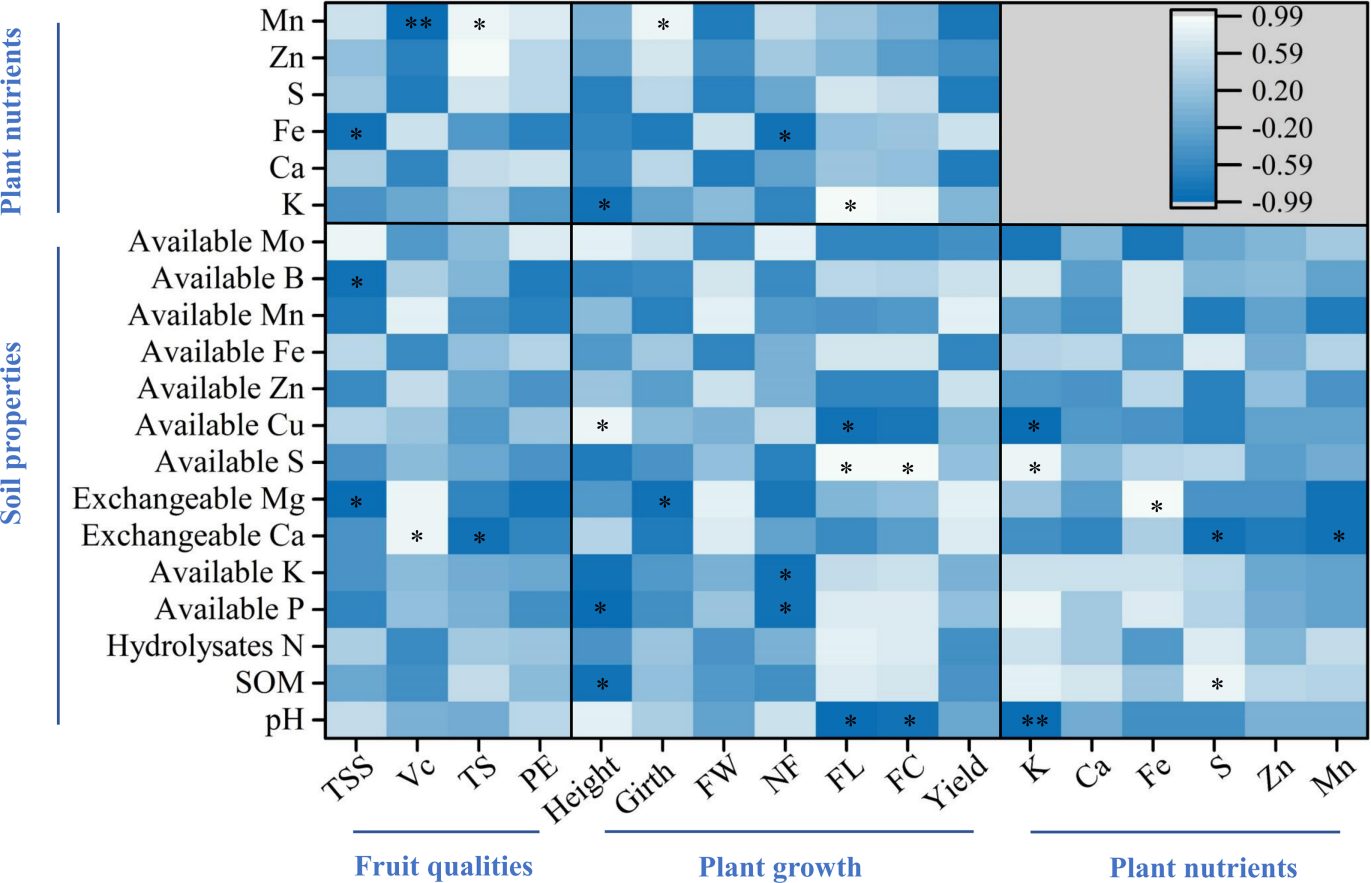
Pearson correlation analyses among soil parameters, key plant nutrients, and fruit quality and yield. The asterisks ^*^ and ^*^^*^ indicate significant correlations at the 0.05 and 0.01 levels, respectively. Plant nutrients represent the accumulation of nutrients per plant. TSS, Vc, TS, and PE represent the percent of total soluble solids, ascorbic acid, total sugar, and proportion of edible fruits, respectively. FW, NF, FL, and FC represent finger weight, number of fingers, finger length, and finger circumference, respectively.

For the fruit qualities, the percent of total soluble solids was significantly and negatively correlated with soil exchangeable Mg and B, and plant Fe, while both ascorbic acid and total sugar were correlated with soil exchangeable Ca and plant Mn (*p* < 0.05, Fig. 6). Regarding plant growth, plant height was correlated with SOM, available P and Cu, and plant K, while plant girth was correlated with soil exchangeable Mg and plant Mn (*p* < 0.05, Fig. 6). Furthermore, finger characteristics (number, length, and circumference) were related to soil pH, available P, K, S, Cu, and plant Fe and K (*p* < 0.05, Fig. 6). For the key plant nutrients, plant K was significantly correlated with soil available S, Cu, and pH, and a significant positive correlation was noted between plant Fe and soil exchangeable Mg (*p* < 0.05, Fig. 6). In addition, both plant S and Mn were negatively related to soil exchangeable Ca, whereas plant S was significantly positively correlated with SOM (*p* < 0.05, Fig. 6).

**Table 4:** Nutrient budgets in the plant crops of different banana varieties.

Organs	Varieties	N^a^	P^a^	K^a^	Ca^a^	Mg^a^	S^a^	Fe^b^	Mn^b^	Cu^b^	Zn^b^	B^b^	Mo^b^	References
Leaves	Plantain	59.6	5.0	47.8	32.2	14.1	5.4	2346.4	8724.2	25.3	143.1	62.6	1.0	Yang et al. (2013)
	Baxi	31.4	2.2	43.3	18.6	6.8	4.9	22.7	611.5	n.a.	12.5	24.3	n.a.	Yao et al. (2005)
	Williams B6	41.7	8.5	64.0	n.a.	n.a.	n.a.	n.a.	n.a.	n.a.	n.a.	n.a.	n.a.	Zhang et al. (2019a)
	Zhongjiao No.9	31.9	2.6	29.2	27.2	7.9	2.2	234.4	4590.7	12.7	33.0	22.1	0.2	This study
Pseudostems	Plantain	46.7	5.2	247.3	41.5	23.1	5.1	686.7	5376.2	53.7	143.0	85.1	1.1	Yang et al. (2013)
	Baxi	26.5	1.5	144.2	21.8	10.8	1.3	154.2	177.1	n.a.	96.4	21.7	n.a.	Yao et al. (2005)
	Williams B6	23.3	2.3	126.3	n.a.	n.a.	n.a.	n.a.	n.a.	n.a.	n.a.	n.a.	n.a.	Zhang et al. (2019a)
	Zhongjiao No.9	18.6	2.3	125.7	21.7	14.0	1.5	576.8	1104.1	16.6	22.5	28.0	0.5	This study
Corms	Plantain	15.0	1.4	88.8	9.8	9.5	1.9	1345.6	1127.5	15.7	206.9	27.8	0.9	Yang et al. (2013)
	Baxi	10.5	0.5	40.8	3.7	5.4	0.4	739.9	109.3	n.a.	45.1	6.2	n.a.	Yao et al. (2005)
	Williams B6	23.8	3.1	137.5	n.a.	n.a.	n.a.	n.a.	n.a.	n.a.	n.a.	n.a.	n.a.	Zhang et al. (2019a)
	Zhongjiao No.9	11.2	1.0	70.3	6.7	7.3	0.5	528.1	682.5	11.2	103.7	11.2	0.2	This study
Roots	Plantain	4.9	0.3	25.6	5.4	2.1	0.4	1639.7	189.9	5.1	15.1	8.6	0.3	Yang et al. (2013)
	Williams B6	2.1	0.2	10.2	n.a.	n.a.	n.a.	n.a.	n.a.	n.a.	n.a.	n.a.	n.a.	Zhang et al. (2019a)
	Zhongjiao No.9	1.4	0.1	10.1	0.8	0.6	0.1	228.8	52.0	1.7	3.9	1.5	0.0	This study
Bunches	Plantain	2.6	0.6	15.1	1.3	0.4	0.5	27.7	142.5	0.8	8.8	4.3	0.2	Yang et al. (2013)
	Zhongjiao No.9	3.8	0.7	20.3	0.8	0.4	0.6	19.8	64.7	0.9	4.6	6.3	0.1	This study
Fingers	Plantain	38.2	6.8	97.1	28.1	5.5	3.3	604.4	582.6	51.7	90.8	23.8	0.7	Yang et al. (2013)
	Baxi	47.3	5.7	109.4	4.6	5.8	2.9	19.1	196.4	n.a.	39.1	37.6	n.a.	Yao et al. (2005)
	Williams B6	42.3	4.4	94.8	n.a.	n.a.	n.a.	n.a.	n.a.	n.a.	n.a.	n.a.	n.a.	Zhang et al. (2019a)
Zhongjiao No.9	18.2	4.7	72.7	0.9	3.6	1.2	43.2	90.0	17.8	19.9	24.6	0.6	This study

**Notes.**

a g plant^−1^.

b mg plant^−1^.

n.a. data not available

**Table 5:** The uptake of nutrients in different banana varieties when the yield reached 60 t ha^−1^.

Varieties	N^a^	P^a^	K^a^	Ca^a^	Mg^a^	S^a^	Fe^a^	Mn^a^	Cu^b^	Zn^b^	B^b^	Mo^b^	References
Plantain	374.4	43.9	1146.1	260.9	121.8	37.3	11.6	36.9	340.2	1368.9	470.2	9.0	Yang et al. (2013)
Baxi	275.3	24.6	900.0	151.2	73.2	23.9	2.1	2.9	n.a.	435.6	228.6	n.a.	Yao et al. (2005)
Zhongjiao No.9	175.9	23.6	668.2	120.0	69.6	12.4	2.9	13.7	124.4	385.8	193.3	3.5	This study

**Notes.**

a kg ha^−1^.

b g ha^−1^.

n.a. data not available

## Discussion

### Plant growth

In this study, the biomass of Zhongjiao No. 9 was mainly distributed in the fingers of mother plants at harvest, with the second highest amount of biomass measured in the pseudostems; the biomass were similar in the leaves, pseudostems, and corms of daughter plants, corroborating the results obtained for Williams B6 (Zhang et al., 2019b). The Zhongjiao No. 9 plants were taller and thicker than other banana varieties, such as Virupakshi (*Musa* spp. AAB) (Kavino et al., 2010), Neypoovan (syn Elakki Bale AB) (Mohandas et al., 2010), Dwarf Cavendish (*Musa paradisiaca* L.) (Alvarez et al., 2001; Bajpai & Saxena, 2017), Red banana (*Musa* AAA) (Humajahan et al., 2018), Grand Naine (*Musa spp.*) (Prakash, Shyam & Prasad, 2019), and Williams B6 (Zhang et al., 2019a). In addition, there were more, longer, stronger, and heavier fingers observed in Zhongjiao No. 9 than that in Virupakshi (Kavino et al., 2010), Dwarf Cavendish (Alvarez et al., 2001), *Nendran* (Bashma et al., 2019), Grand Naine (Prakash, Shyam & Prasad, 2019), or four Indonesian banana cultivars (Pisang Berlin (*Musa* AA), Ambon Hijau (*Musa* AAA), Raja Bandung (*Musa* ABB), and Kepok (*Musa* ABB)) (Hapsari & Lestari, 2016). Although these studies were carried out in different climates and under different fertilization and irrigation, they still provide a perspective for comparative studies.

### Fruit yield and quality

In this study, the yield of Zhongjiao No. 9 was similar to that of Williams B6 (Zhang et al., 2019a), but the yield of Zhongjiao No. 9 was significantly superior to the low yields of East African highland bananas (Ndabamenye et al., 2013a), plantain (Yang et al., 2013), and *Nendran* (Bashma et al., 2019). Due to its high resistance to Fusarium wilt and relatively high yield, the prospect of planting Zhongjiao No. 9 has shown considerable promise.

It is evident from the data that lower total soluble solids and ascorbic acid levels and higher total sugar levels were observed in Zhongjiao No. 9 fruits than those of Virupakshi (Kavino et al., 2010), Grand Naine (Sandhya et al., 2018), and four Indonesian bananas (Hapsari & Lestari, 2016); this result may be explained by the soil exchangeable Ca and plant Mn concentrations (Fig. 6). The negative correlation observed between ascorbic acid and plant Mn was similar to those found in previous studies of tomato fruit (Muzolf-Panek, Kleiber & Kaczmarek, 2017) and pepper (Tewari, Kumar & Sharma, 2006). Elevated Mn concentrations may directly inhibit ascorbate due to three possible non-exclusive explanations: (i) oxidative stress induction in plants, (ii) modification of the regenerative cycle of oxidized forms of ascorbate, and (iii) maintenance of the redox status of plant cells (Tewari, Kumar & Sharma, 2006; Muzolf-Panek, Kleiber & Kaczmarek, 2017). Furthermore, a significant positive correlation was noted between ascorbic acid and soil exchangeable Ca (Fig. 6), and similar relationships were also found a strawberry plantation (Singh, Sharma & Tyagi, 2007). In the present study, the Ca concentration was very low in both the roots and fruits of Zhongjiao No. 9. Previous studies have provided evidence indicating that the involvement of Ca slows down ripening and senescence processes in other fruits (Ferguson, 1984; Singh, Sharma & Tyagi, 2007). We also observed that the fruit of Zhongjiao No. 9 was prone to browning during the ripening processes. The very low levels of Ca concentration measured in the fruit confirmed these processes. In addition, the P, K, Ca, Mg, Cu, Zn, and Mn levels of the edible portion of Zhongjiao No. 9 were significantly higher than those reported in the literature not from particular named cultivars (Zahir, Naqvi & Uddin, 2009; Ismail et al., 2011; Hapsari & Lestari, 2016); thus, the fruit of Zhongjiao No. 9 could provide better dietary minerals than other banana cultivars.

### Plant nutrients

The uptake of nutrients was in the order of K >N >Ca >Mg >P >Mn >S >Fe >Zn >B >Cu >Mo in Zhongjiao No. 9 plants, corroborating the results of some previous studies (Yao et al., 2005; Moreira & Fageria, 2009; Yang et al., 2013). Compared with plantain and Baxi at a high yield of 60 t ha^−1^, most nutrients were stored less in Zhongjiao No. 9 (Table 4). As a banana plantation produces more, nutrient removal also increases (López, 1999; Ndabamenye et al., 2013a). The less nutrient uptakes in Zhongjiao No. 9 at the same yield suggested that this new cultivar needs few nutrient input and has a high nutrient utilization efficiency.

As noted by Zhang et al. (2019b), the high nutrient levels measured in corms and pseudostems at harvest favor the growth of suckers in mother plants compared with those at the fruit development stage, while leaf nutrients were allocated to the fruits at the fruit development stage; thus, the leaf nutrient accumulation was lower at harvest. Similarly, we found that higher Ca, Mg, S, Mn, and/or Fe concentrations were observed on the leaves, pseudostems, and/or corms of mother plants than of daughter plants. It is generally accepted that N, P, K, Ca, and Mn are the most limiting nutrients for banana growth and yield (Turner & Barkus, 1983; Smithson et al., 2004; Nyombi et al., 2010; Wairegi & Asten, 2010; Ndabamenye et al., 2013a). The current study showed that plant K, Ca, and Fe were important for both mother plants and daughter plants growth, while S, Zn, and Mn were especially important for mother plants, and N, P, and B were important for daughter plants (Fig. 4). In addition, these key nutrients were high association with the soil pH, SOM, available S, Cu, and Fe, and exchangeable Mg and Ca concentrations in the present study (Fig. 6).

Previous studies have suggested that some nutrient elements in banana plants and their associated soils can reduce the severity of plant diseases by increasing disease tolerance and resistance against plant pathogens (Dordas, 2008; Siddiqui et al., 2015; Wu et al., 2020). There were positive correlations observed between the contents of Zn, Mn, Mg, and P in banana and the suppression of wilt disease severity (Wu et al., 2020). In present study, the nutrient uptake ratio of N:P:K:Ca:Mg:S were 1:0.13:3.86:0.68:0.40:0.07 (Zhongjiao No. 9), 1:0.12:3.12:0.71:0.33:0.10 (plantain), and 1:0.90:3.72:0.55:0.27:0.09 (Baxi). Interestingly, there was a relatively high Mg uptake ratio measured in Zhongjiao No. 9; specifically, high Mg concentrations were measured in the pseudostems. Banana Fusarium wilt is caused by vascular wilt pathogens, which satisfy their nutritional requirements by efficiently acquiring the scarce nutrients available in the xylem sap, by enzymatic digestion of host cell walls, by invading neighboring cells, or by inducing nutrient leakage from surrounding tissues (Divon et al., 2005; Möbius & Hertweck, 2009; Klosterman et al., 2011; Yadeta & Thomma, 2013). High Mg accumulations could enhance plant suppression to Foc4 (Wu et al., 2020). Therefore, the absorption and accumulation of Mg may be a key mechanism related to nutrient element modulation. Further evidence in this regard is that magnesium oxide (MgO) is a promising agent used for the control of Fusarium wilt in both tomato and Arabidopsis thaliana (Imada et al., 2016; Ota et al., 2019; Fujikawa et al., 2020). Further studies are needed in the future to investigate the absorption and accumulation of Mg.

### Implications of findings and research outlook

Maintaining the nutrient levels of crop fields should be considered during the management of banana plantations. Fertilization application should be strictly calculated on the basis of crop demand, and any excessive application should be avoided to prevent environmental problems (Raza et al., 2020). The measured biomass and yield of Zhongjiao No. 9 were higher with lower nutrient uptake compared with other banana varieties, indicating that Zhongjiao No. 9 has a high nutrient utilization efficiency. During plant crop growth, the nutrient uptake ratios of N:P:K:Ca:Mg:S (1:0.13:3.86:0.68:0.40:0.07) could provide information on the nutrient demands of the plants and on fertilization applications. The K, Ca, and S levels in the plants were found to be important for the plant crops (Fig. 4). More attention should be paid to the application of K, Ca, and S fertilizers as well as their influencing factors during plant crop growth. When nutrients are output by fruit, residual nutrients should be recycled. These nutrients from residues could be subsequently available to the root systems and assimilated by daughter plants (Yamaguchi & Araki, 2004; Ndabamenye et al., 2013b). Caution should be taken by maximizing the use of residual input to avoid depleting fields, to ensure the application of reasonable fertilizer replenishment, and to improve the nutrient utilization efficiency and maintain nutrient levels in fields. In fact, the amounts of nutrient removed by harvesting and pruning were not equal to those added by fertilization due to the difference in the utilization ratios of nutrients and the capacity of the soil. In the future, it will be necessary to systematically study the nutrient uptake and utilization capacity of plants and the nutrient absorption characteristics of soils to provide more accurate data to support the nutrient management of Zhongjiao No. 9.

This study explored, for the first time, the growth and nutrient budgets of a Fusarium wilt-resistant banana cultivar. This plant has a high yield in the field as well as resistance to Fusarium wilt. In Fusarium-infested soils, Zhongjiao No. 9 has considerable promise. However, a lack of knowledge about fertilizer management is a large hindrance to the widespread promotion of the Zhongjiao No. 9 planting. Further studies are needed in the future to investigate optimal fertilizer management if this cultivar is widely planted. In addition, further studies should identify and quantify the relationships among nutrient dynamics and diseases, especially the absorption and accumulation of Mg, and further development of disease-resistant varieties is necessary for food security and diversified needs.

## Conclusions

The prospect of planting Zhongjiao No. 9 has shown considerable promise. Our work improves the current understanding of the plant growth, yield, fruit quality, and nutrient budgets of Zhongjiao No. 9. The current study showed that the K, Ca, and Fe levels in plants were important for both mother and daughter plant growth, while the S, Zn, and Mn levels in the mother plants, and the N, P, and B levels in daughter plants were important for plant growth. These key nutrients were highly associated with the soil pH, SOM, available S, Cu, and Fe, and exchangeable Mg and Ca concentrations. The absorption and accumulation of Mg may be a key mechanism related to nutrient element modulation. Furthermore, the nutrient uptake ratios of N:P:K:Ca:Mg:S provide information on nutrient demand and fertilization application.

##  Supplemental Information

10.7717/peerj.11141/supp-1 Supplemental Information 1 Fresh weight and nutrient concentrations.

## Additional Information and Declarations

### Data Availability

The following information was supplied regarding data availability: Raw data are available in the Supplemental Files.
